# Visualizing youth sports specialization and injury risk: A novel application of swimmer plots

**DOI:** 10.1371/journal.pone.0352327

**Published:** 2026-06-25

**Authors:** Julie Agel, Nicholas Edwards, Bradley Nelson, Todd Rockwood

**Affiliations:** 1 Department of Orthopedics, University of Minnesota, Minneapolis, Minnesota, United States of America; 2 Division of Health Policy and Management, University of Minnesota, Minneapolis, Minnesota, United States of America; Universidade do Porto, PORTUGAL

## Abstract

**Background:**

As research on sport participation in youth and the impact of sport specialization on injury increases, the value of a standardization of data variables and an easily interpretable way of visualizing the data will be demonstrated with improved quality and comparability of future findings.

**Purpose:**

Using simulated sport participation data from the perspectives of chronological age and years of participation, the utility and flexibility provided by swimmer plots is a novel method for visualization of data to assess the impact of single versus multiple sport participation on the occurrence of injury.

**Methods:**

Using recall survey data, a simulated dataset based on chronological age as well as years of participation was created to allow for differing visualizations of the impact of single vs multiple sport participation on the occurrence of injury using swimmer plots.

**Results:**

Characterization and presentation of sports history are deceptively complex and the reliance on tabular and simple graphics fail to provide a comprehensive picture. Collecting a core data set, allowing for multiple denominators such as chronological age and years of participation along with using swimmer plots as an effective means of visually presenting the data allow for a more robust and multi-faceted approach to the understanding of the impact of youth early sport specialization on injury risk. Demonstrated are examples of injury occurrence as it relates to chronological age of participation as well as years of participation. Figures demonstrating differences in injury rates based on number of sports participated in at various ages are also provided.

**Conclusion:**

Swimmer plots allow for visualization of data from multiple perspectives which facilitates a comprehensive understanding of the complex, overlapping factors potentially impacting injury in youth sports. Visualizing more than one perspective allows for a more complete picture of the factors involved.

## Introduction

As the complexity of the data required to answer the research question, “Does youth early sport specialization impact injury risk?” is better clarified there is an ongoing effort to strengthen the methodology used to assess sport specialization and its impact on injury risk [1–3]. This effort includes solidifying a definition for sport specialization that accounts for various permutations of participation. Current commonly used definitions include “Intentional and focused participation in a single sport for a majority of the year that restricts opportunities for engagement in other sports and activities” or “Participation in intensive training and/or competition in organized sports greater than 8 months per year” [4–6]. As Güllich comments [7] these dichotomous or categorical labeling ignore many potentially relevant factors. An athlete could choose to play one sport year-round or multiple sports year-round but on multiple teams within a season. Specialization may also be self-defined; the athlete participates in several sports but consider themselves to be specializing in one of those sports. A broader collection of variables required to confidently define and track the impact of sport specialization on injury in a longitudinal fashion is central to generating useful research. There is also the recognition that the answers may vary based on the sport being played. Sports requiring flexibility may require different athletic components versus sports requiring strength.

The development of a survey platform and review of unpublished pilot data by our research team to evaluate the impact of youth early sport specialization on injury risk brought into focus the importance of measuring the impact of participation from the perspectives of both chronological age as well as time spent in sport. The variables required to accurately investigate this concern have evolved from time spent in a sport (exposure) to include the intensity level of participation, the number of teams, other sport activities, injury specificity, and time loss.

This paper focuses on the utility of swimmer plots as the primary method for data visualization of sports history and related participation and injury events. It derives from lessons learned from multiple developmental trial survey administrations in youth and professional athletes by our research team in preparation to start a longitudinal prospective project. It demonstrates the utility of a proposed core dataset which contains the perspectives of chronological age as well as time spent in sport participation. The flexibility of the swimmer plots provides a visual framework for presentation and evaluation of data with multiple techniques to stratify results (individual, population and teams/league) and identification of specific events (i.e., date of specialization, injury specifics) within the context of an entire athletic career.

## Materials and methods

As preliminary steps to a longitudinal study of early youth sports participation and its impact on injury an initial survey was administered via paper and pencil to a group of professional athletes asking for historical participation data. This survey had institutional IRB approval and respondents had an IRB approved information sheet as part of the survey. No identifying information about any respondent was collected. A waiver of consent was granted by the IRB. This recall survey formed the basis for the development of a core set of variables [8] which included the following:

1. Current age of the athlete.
2. Age at which they started pre-competitive play (free or deliberate) in a sport(s).
3. Age at which they started competing in a sport(s).
4. Age at which they specialized in a sport.
5. Age at which they started competing at the elite level in the sport.
6. Age at which they quit a sport.
7. Age(s) at which injuries that prohibited participation in the sport occurred and time loss.

The age variables were used for the calculation of the secondary variables tied to duration of participation:

1. Years of participating in the sport.
2. Years of competition in the sport.
3. Years of specialization in the sport.
4. Years competing at the elite level in the sport.
5. Years of participation relative to injuries sustained while playing the sport and time loss.

Within the survey, injury was defined as 12 weeks or more of missed participation.

Data collection for the baseline sample on which the simulated data was based occurred in March of 2017.

Sample size estimation and justification:

This is simulation data meant to illustrate the utility of swimmer plot in the presentation of sports history data between populations and leagues.

Using data gathered from the initial recall survey of 74 athletes who had already attained elite level participation, a simulation dataset of 1200 cases was created in Excel® Microsoft. This dataset was created to demonstrate how data aggregated in different ways (population, league v. conference) can be visualized and compared using swimmer plots to evaluate sports participation history, specialization, and injury.

This simulation data was aggregated into 12 age groupings, based on age starting participation in what was considered the target (or ultimate sport specialized in) sport (3–14 yrs) or 10 groupings based on years of participation (each decile represents between 90–122 cases). Participation in this simulation is characterized as having four levels of participation: free/deliberate play (non-competitive, casual, pick-up, playground type activities, competitive play which would be when scoring, winning and losing comes into play, specialization, and elite play which would be considered professional. Minimum and maximum values from the original recall survey data for each of the age groups allowed for delineating the average age of transition to competitive participation, transition to specialized participation, transition to elite level*,* current age. These calculated values served as the upper and lower bounds for creation of random ages for each of the same fields in the simulated data. These were then converted into years of participation to demonstrate duration of exposure at each level of participation. The probability of any given simulated case having an injury was based on the rate of injury in the existing recall dataset. For those randomly assigned an injury the minimum and maximum and ranges were set based on the existing data using the assignment of age at time of injury. Given the complexity of generating data for the history of multi-sport participation, the values generated were informed by using parameters derived from the recall surveys conducted in elite athletes

Programming for the creation of the swimmer plots was done using SAS (© v 9.4) (supplemental material). In evaluating the data presented in the swimmer plots the length of each segment represents the denominator (in these examples of either chronological age or years of participation) and the transition line between levels of play is the average point of change for the population and the bar inside represents the 95% confidence interval. To fully explore the utility of swimmer plots the four levels of participation shown here are representative of actual participation trajectories but could be changed to reflect other pathways based on the research being undertaken.

The figures presented here show the data grouped for a population such as a team or league of players; in these examples’ simulation data representing 1200 athletes with the height of the decile reflective of the number of athletes within each decile. The figures utilize two denominators to frame the data for presentation and allow for complimentary perspectives: chronological age and time spent in the sport with overlays for injury occurrence and multiple sport participation. The swimmer plots presented form the basis of an analysis plan for a prospective longitudinal cohort project.

## Results

**Fig 1:**
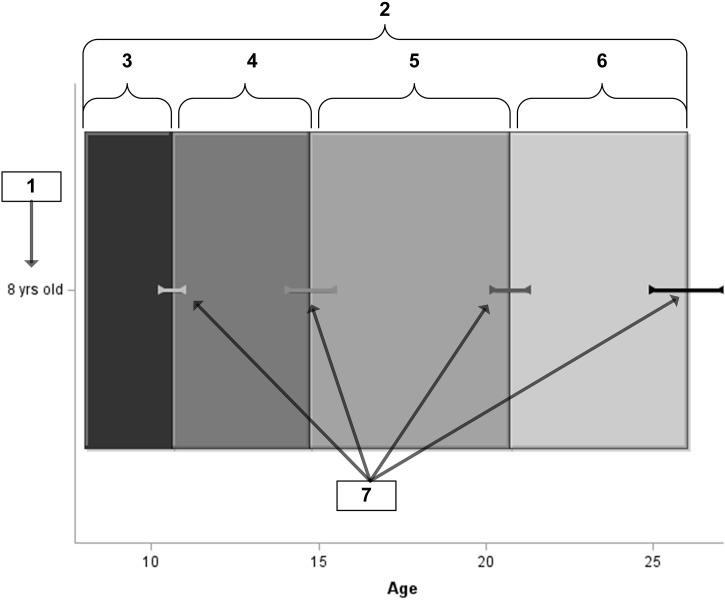
Illustrative example of the core components in a Swimmer Plot. 1 is the y-axis representing the unit of measurement specific to that Fig 2 is the x-axis, in this example chronological age. 3 is the ages spent in free/deliberate play (Black). 4 is the ages spent in competitive play (Dark gray). 5 is the ages spent in specialized play (light gray), 6 is the ages spent in professional play (lightest gray), 7 is the 95% Confidence bar representing the average age of transition between levels of play.

Fig 1 is a breakdown of the swimmer plots as used in the examples presented. The changes in shading within each line or decile represent different levels of participation (e.g., free/deliberate play to professional). Within each line confidence bars (95% confidence intervals for average) indicate the range of transition from one level of competition to another.

**Fig 2:**
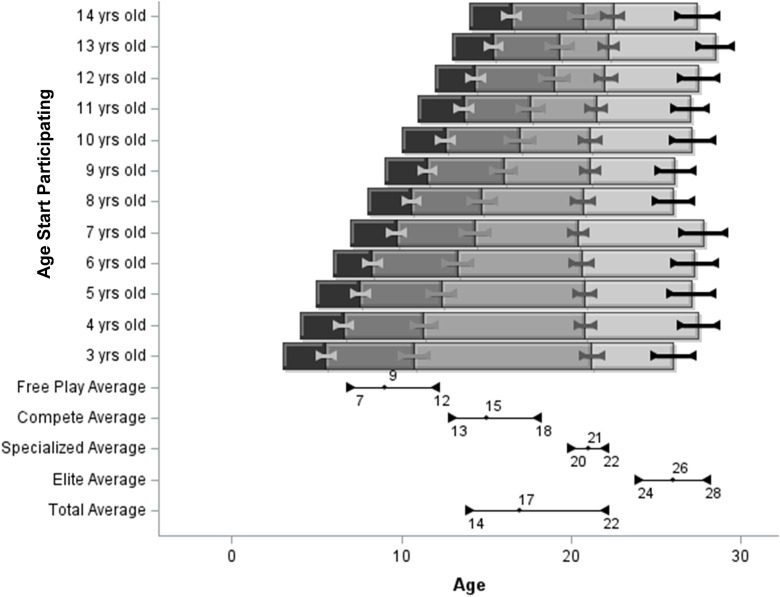
Simulated data characterization of career history by chronological age. Black (left) is the average age of participation in a sport prior to specialization. Dark gray (2nd left) segment is average age during competitive play. Light gray (3rd left) is average age of participation in a sport once specialized. Lightest gray (right) is average age of participation at the elite level. 95% confidence intervals represent average ages around the transition point between segments. Confidence Bars (left): average age of transition from free play to competitive participation (1^st^ from left), average age of transition from competitive participation to specialized play. (2nd from left): third average age of transition from specialized to elite participation. (3rd from left): last segment is ages playing at the elite level (light gray) Average current age of simulated population, bottom bar is the overall average and 95% confidence interval of ages playing sport (total average). Footnote: Figure is sorted in order based on age starting participation in the sport.

Fig 2 demonstrates four distinct aspects of participation based on the ages that participation in the target sport began: free/deliberate play, competition, specialization, and elite play. The Y-Axis is based on grouping data based on age starting to participate in the sport. The length of each color segment represents the range of ages that athletes spend in each level of play. The confidence bars between each level of play represent the 95% CI for the average age of transition across levels of play within each decile grouping. Regardless of the age that participation in the sport began Fig 2 demonstrates the transition from free play to competitive participation occurred on average at 9 (95% confidence interval = 7,12) years of age, the transition from competitive participation to specialization in a sport at 15 (95% confidence interval = 13,18) years of age, the transition from specialized participation to elite participation occurred at 21 (95% confidence interval = 20,22) years of age. For the total study population, the average current age is 26 (95% confidence interval = 24,28).

To assess the impact of chronological age on participation in sport Fig 2 allows for visualization of the variation which occurs in age at which competitive participation in the sport began. Fig 2 shows that regardless of the age starting the target sport the years spent in free play and competitive participation are uniform across the entire population. The pattern for specialized participation shows that within this dataset age starting the sport has an impact on how long they spend in specialized participation, with those starting younger spending more time in competitive participation while those who started the sport at an older age moved to specialized participation sooner.

**Fig 3:**
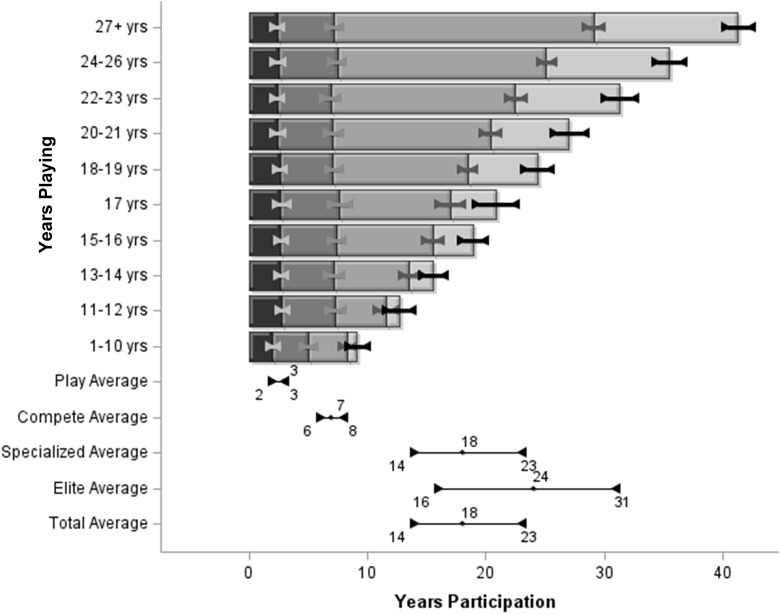
Simulated data characterization of career history based on years of participation. Black (left) is average years of participation in a sport prior to specialization. Dark gray (2nd left) segment is average years of competitive participation. Light gray (3rd left) is average years of participation in a sport once specialized. Lightest gray (right) is elite level participation. 95% confidence intervals represent average years around the transition point of each segment. Confidence Bars: (left) transition into specialization. (center) transition into elite. (right) total years of participation. The lines at the bottom are the total population averages around the transition points in career history. Footnote: Figure is sorted by cumulative years of participation.

Fig 3 visualizes the years participating in the target sport by level of participation. The x-axis is the grouping of players into deciles. The x-axis identifies the years of participation across different levels of play. Fig 3 identifies that those who have participated in the target sport for less than 10 years have spent less time in each level of play when compared to those who have participated more than 11 years. Fig 3 demonstrates that time in specialized participation increases as total years in sport increases and time spent at the elite level ties directly to years participating. The average years of transition spent in participation from free/deliberate play to competitive participation is 3 years (95% confidence interval = 2, 3), from competitive to specialized participation is 7 (95% confidence interval = 6, 8), specialized participation to elite participation is 18 (95% confidence interval = 14, 23), total years of play at elite level on average is 24 years (95% confidence interval = 16,31). The final line is average total number of years playing the sport 18 (ci 14–23.)

Fig 2 (age based) and 3 (years played) illustrate contrasting points of view relative to data presentation and evaluation. Fig 2 allows for visualization of the data from the developmental point of view in which age is the organizing principle while Fig 3 provides visualization of the data from the point of view in which years participated. is the organizing principle. Comparing and contrasting these two figures demonstrates the ability of swimmer’s plots to illustrate different characterizations of sports history clearly and effectively both at the individual and group level.

**Fig 4:**
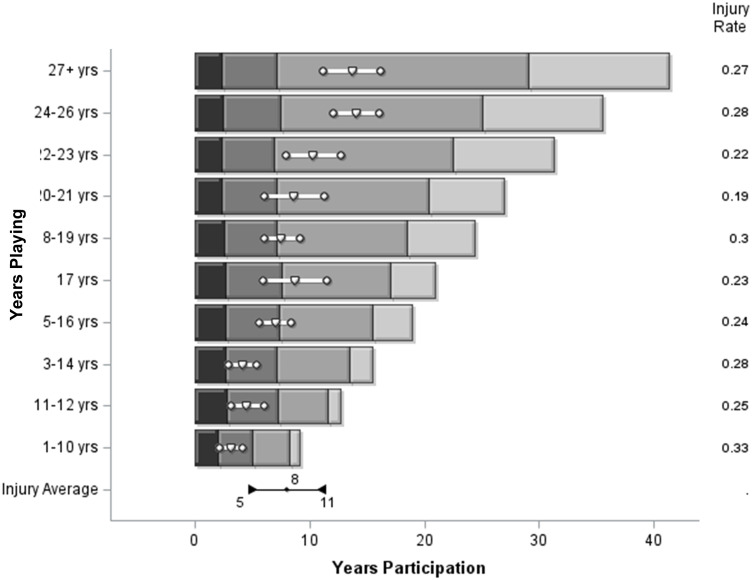
Simulated data characterization with overlay of years to 1st injury on career history based on years of participation. Black (left) is average years of participation in a sport prior to specialization. Dark gray (2nd left) segment is average years of competitive participation. Light gray (3rd left) is average years of participation in a sport once specialized. Lightest gray (right) is average years of participation at the elite level. Confidence Bar is the 95% confidence interval around average years of participation at which first injury occurred. Secondary y-axis is injury rate within each decile.

Fig 4 presents the simulated injury overlay on years participated with the confidence bar representing the 95% confidence interval around the average age of occurrence of first injury. While the average years participated in the sport prior to injury is 8 (95% confidence interval = 5,11), those who have played the sport 14 years or less have injuries concentrated in the pre-specialization period, compared to those who have played the sport 15–23 years, in which injuries tend to span the pre and specialized portion of their career. For those with long participation (24 + years) injuries occur late in the specialized prior to the elite portion of their history. Integrated into the graphic the column of numbers on the right provides the rate of injury within each year of participation group.

**Fig 5:**
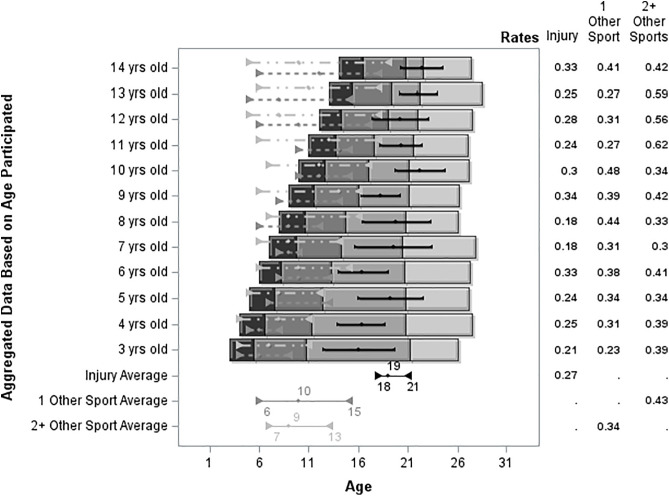
Simulated data characterization with overlay of multi-sport participation history on career history based on chronological age. Black (Left) is the average age of participation in a sport prior to specialization. Dark gray (2nd left) segment is average age of competitive play. Light gray (3rd left) is the average age of participation in a sport once specialized. Lightest gray is the average age of participation at the elite level participation (right). The gray lines within each primary bar represents the CI for 1 other sport (lighter) and multiple other sports (darker) and the diamond is the average ages playing multiple sports. The two dark lines at the bottom are the 95 CI and means for the total population. Secondary y-axis is the injury rate, the rate playing one other sport or 2 or more other sports. The solid black line in the middle of the bars is the mean and 95% CI for first injury occurrence.

Evaluating multi-sport history Fig 5 overlays multi-sport history by age and occurrence of 1st injury on career in the target sport. The graphic shows that those who started the target sport very young (<5 years old) on average added additional sports after the age they started the target sport. For those that came to the target sport later in life they, on average, started playing other sports well before starting the target sport (this is based on the lighter and darker lines found within each of the primary bars in the graphic). The average age of transition (diamond symbol) occurs about midway through the pre-specialization competitive portion of their participation ending at the point of specialization and the length of line is the 95% confidence interval around that average. For those that played only one other sport, on average they started playing the additional sport at 9 (95% confidence interval = 7,13) and those that played 2 + other sports began those sports at 10 (95% confidence interval = 6,15). Given that this is population not individual data those individuals who specialized at an older age the 95% confidence intervals on single/multi-sport participation will extend into the specialization segment in the graph.

## Discussion

The purpose of this paper is to demonstrate the novel usage of swimmer plots to present sports participation data from multiple perspectives with flexibility based on differing size populations. They can be used to compare participation profiles across different sports as risk factors may not be the same by position (pitcher versus 1^st^ baseman in baseball) or across sports (Gymnastics versus Ice Hockey). Within this presentation we have selected to use chronological age and years participating as the underlying metric used in creating the swimmer plots, but this can be modified for any setting and any unit of measurement (i.e., age/years to 1^st^ injury, number of different sports participated in). The presentation of these simulations demonstrates the utility and flexibility of swimmer plots to summarize data from different perspectives, such as a complete participation history, in which each segment within each decile represents a level of play (free play, competitive play, specialized play, elite level play). A fundamental benefit of using swimmer plots is to easily visually transition between different methods of data evaluation. The plots facilitate the ability to organize and structure the data such that it can be compared for evaluation based upon different organizing principles (age versus years participated). One of the fundamental advantages of swimmer plots is the ability to evaluate and present other related events based on the historical description of sports participation history, e.g., a focus on exposure (years participated) and injury within the context of pre/specialization.

Heat maps, as suggested by Larson et al. [9] allow for two-dimensional visual presentation of data. while swimmer plots allow for multiple visual overlays of factors that may play a role in determining the impact of sports specialization on injury.

The dual perspective of chronological age and time spent participating demonstrate the importance of variable perspectives in data analysis. A tennis player who starts playing competitive tennis at 6 years of age is considered specialized, and plays for 6 years is different from a tennis player who starts playing at age 16 for 6 years and is considered specialized. Fig 3 shows the mean individual player overlap of number of years spent participating prior to specialization and years spent in participation once specialized are equivalent but represent different levels of participation intensity and potentially different levels of risk for injury. The variables identified in earlier work [8] and used in the examples within this work allow for standardized reporting of multiple sport participation including the potential transition for specialization in one sport to specialization in another sport over time. They also allow for the dropping out of sports, a factor often overlooked in analysis. By utilizing visualization methods that allow for the integration of a variety of variables, the picture presented of longitudinal sports participation becomes more complete. With the ability to integrate more variables into the analysis plan researchers can move closer to understanding the impact of youth early sport specialization on injury.

## Supporting information

S1 Supplemental Materials Example of SAS code to create swimmer plots. (DOCX)

S2 Article data Raw data file. (XLSX)

## References

[1] Agel J, Post E. Early sport specialization. J Bone Joint Surg Am. 2021;103(20):1948–57. 34375322 10.2106/JBJS.21.00018

[2] Kliethermes SA, Marshall SW, LaBella CR, Watson AM, Brenner JS, Nagle KB, et al. Defining a research agenda for youth sport specialization in the United States: the AMSSM Youth early sport specialization summit. Clin J Sport Med. 2021;31(2):103–12. doi: 10.1097/JSM.0000000000000900 33587486

[3] Nielsen RO, Shrier I, Casals M, Nettel-Aguirre A, Møller M, Bolling C, et al. Statement on methods in sport injury research from the 1st METHODS MATTER Meeting, Copenhagen, 2019. Br J Sports Med. 2020;54(15):941. doi: 10.1136/bjsports-2019-101323 32371524 PMC7392492

[4] Bell DR, Snedden TR, Biese KM, Nelson E, Watson AM, Brooks A, et al. Consensus definition of sport specialization in youth athletes using a Delphi approach. J Athl Train. 2021;56(11):1239–51. doi: 10.4085/1062-6050-0725.20 33787895 PMC8582622

[5] Jayanthi N, Kliethermes SA, Côté J. Youth sport specialisation: the need for an evidence-based definition. Br J Sports Med. 2020;54(4):196–7. doi: 10.1136/bjsports-2019-101256 31857339

[6] Nelson EO, Bell D, Kliethermes S, Biese KM, Renner MN, Srygler EC, et al. Item bank development and content validation of a youth sport specialization assessment. JOSPT Open. 2025;3(1):22–9. doi: 10.2519/josptopen.2024.0065

[7] Güllich A, Macnamara BN, Barth M, Hambrick DZ. Further muddying the waters? A comment on Bell et al’s 2021 definition of youth sport specialization. J Athl Train. 2021;56(11):1252–4. doi: 10.4085/1062-6050-1010-21 34752629 PMC8582632

[8] Rockwood T, Edwards NM, Nelson B, Agel J. Evaluating the impact of youth early sport specialization on injury: an evolution in measurement. Health Serv Res Manag Epidemiol. 2023;10:23333928231176207. 37251699 10.1177/23333928231176207 PMC10209588

[9] Larson HK, Young BW, McHugh T-LF, Rodgers WM. Visual representations of single- and multi-sport participation in a youth swimming sample: implications for definitions and discussions of early specialization. PLoS One. 2023;18(9):e0292038. doi: 10.1371/journal.pone.0292038 37756317 PMC10530013

