# Supplementary material for: Visualizing youth sports specialization and injury risk: A novel application of swimmer plots
Source: PLoS One. 2026 Jun 25;21(6):e0352327. doi: 10.1371/journal.pone.0352327 (PMC13298933; doi:10.1371/journal.pone.0352327)
Supplement: S1 Supplemental Materials — (DOCX) [file pone.0352327.s001.docx]

**Supplemental Materials**

Example of SAS code to create swimmer plots:

Step 1: Core Lanes Sport Participation History


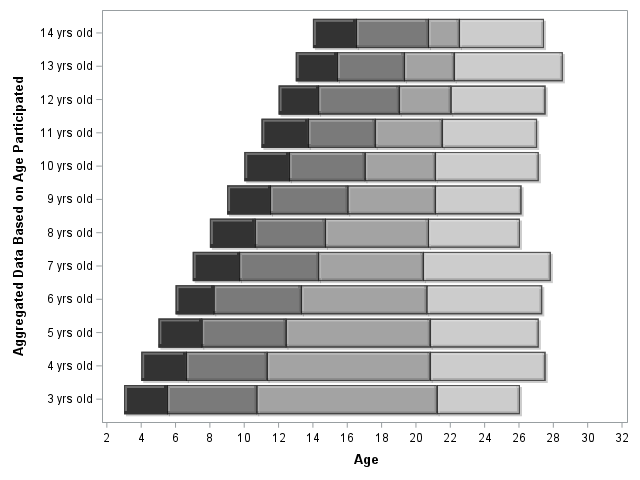


**ods graphics on /noborder;**

**proc sgplot data=work.sgroup CYCLEATTRS noautolegend ;;**

***core lines by age start;**

**highlow**

**y=GROUPAGESTART1 LOW=GROUPAGESTART1 HIGH=AGE_comp_mean**

**/type=bar fillattrs=(color=Black) dataskin=cRISP**

**LINEATTRs=(color=black) transparency=.2;**

**highlow**

**y=GROUPAGESTART1 LOW=AGE_comp_mean HIGH=AGE_spec_mean**

**/type=bar fillattrs=(color=dagr) dataskin=cRISP**

**LINEATTRs=(color=black) transparency=.2;**

**highlow**

**y=GROUPAGESTART1 LOW=AGE_spec_mean HIGH=AGE_ELITE_MEAN**

**/type=bar fillattrs=(color=megr) dataskin=cRISP**

**LINEATTRs=(color=ligr) transparency=.2;**

**Highlow**

**y=GROUPAGESTART1 LOW=AGE_ELITE_MEAN HIGH=AGE_CAGE_MEAN**

**/type=bar fillattrs=(color=ligr) dataskin=cRISP**

**LINEATTRs=(color=ligr) transparency=.2;**

***Format Axis;**

**xAXIS VALUES=(2 to 32 BY 2)**

**label='Age' labelattrs=(family=arial weight=bold);;**

**yaxis values=(3 to 14 by 1)**

**Label="Aggregated Data Based on Age Participated"**

**labelattrs=(family=arial weight=bold);;**

**run;**

Step 2: Add Mean and 95% Confidence Limits (bold code is what adds this to graphic). For other sport history (1 other sport (light gray), 2+ other sports (medium gray)) and injury (black)


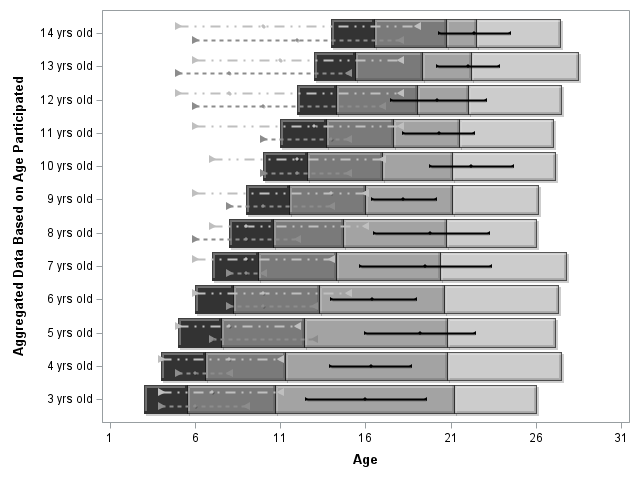


ods graphics on /noborder;

**proc** **sgplot** data=work.sgroup CYCLEATTRS noautolegend ;;

*core lanes by age start code above included;

*additional code to add mean/95ci;

***ci lines: injury;**

**highlow**

**y=groupagestart low=age_inj_lcl high=age_inj_ucl**

**/lineattrs=(pattern=solid color=black thickness=2 )**

**dataskin=crisp ;**

**scatter**

**y=GROUPAGESTART x=age_inj_lcl**

**/ markerattrs=(symbol=TriangleRightFilled size=3**

**color=black);**

**scatter**

**y=GROUPAGESTART x=age_inj_ucl**

**/ markerattrs=(symbol=TriangleleftFilled size=3**

**color=black);**

**scatter**

**y=GROUPAGESTART x=age_inj_mean**

**/ markerattrs=(symbol=diamondFilled size=3 color=black);**

***ci lines: single sport;**

**highlow**

**y=ageci low=rate_ss_lcl high=rate_ss_ucl**

**/ y2axis lineattrs=(thickness=2 pattern=shortdash**

**color=megr) ;**

**SCATTER**

**Y=ageci X=rate_ss_lcl**

**/ y2axis FILLEDOUTLINEDMARKERS**

**markerfillattrs=(COLOR=megr) markeroutlineattrs=(COLOR=megr)**

**markerattrs=(symbol=TriangleRightFilled SIZE=8 );**

**SCATTER**

**Y=ageci X=rate_ss_ucl**

**/ y2axis FILLEDOUTLINEDMARKERS**

**markerfillattrs=(COLOR=megr)**

**markeroutlineattrs=(COLOR=megr)**

**markerattrs=(symbol=TriangleLeftFilled SIZE=8 ) ;**

**SCATTER**

**Y=ageci X=rate_ss_mean**

**/ y2axis markerattrs=(symbol=diamondfilled size=3**

**color=megr) dataskin=crisp;**

***ci lines: multi-sport;**

**highlow**

**y=ageci low=rate_ms_lcl high=rate_ms_ucl**

**/ y2axis lineattrs=(thickness=2 pattern= DASHDOTDOT**

**color=ligr) ;**

**SCATTER**

**Y=ageci X=rate_ms_lcl**

**/ y2axis FILLEDOUTLINEDMARKERS**

**markerfillattrs=(COLOR=ligr)**

**markeroutlineattrs=(COLOR=ligr)**

**markerattrs=(symbol=TriangleRightFilled SIZE=8);**

**SCATTER**

**Y=ageci X=rate_ms_ucl**

**/ y2axis FILLEDOUTLINEDMARKERS**

**markerfillattrs=(COLOR=ligr)**

**markeroutlineattrs=(COLOR=ligr)**

**markerattrs=(symbol=TriangleLeftFilled SIZE=8 ) ;**

**SCATTER**

**Y=ageci X=rate_ms_mean**

**/ y2axis markerattrs=(symbol=diamondfilled size=3**

**color=ligr) dataskin=crisp ;**

**xAXIS**

**VALUES=(1 to 35 BY 5) label='Age'**

**labelattrs=(family=arial weight=bold);**

**yaxis values=(3 to 14 by 1)**

**Label="Aggregated Data Based on Age Participated"**

**labelattrs=(family=arial weight=bold);;**

**y2axis values=(3 to 14 by 1) display=none;**

Step 3: Add rates for multi-sport (1 v. 2+) history and injury to Y2Axis.


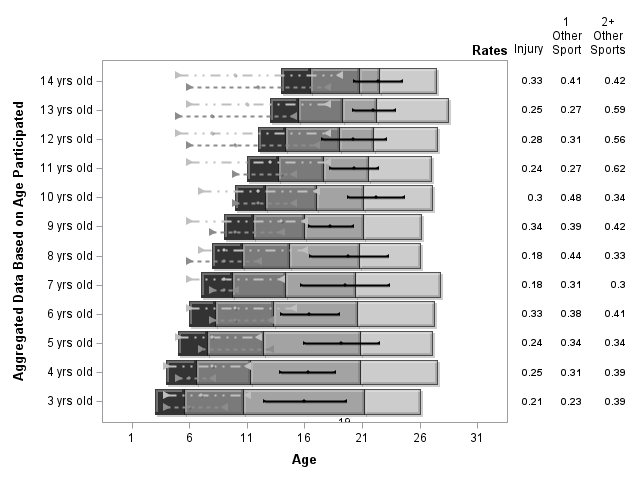


ods graphics on /noborder;

**proc** **sgplot** data=work.sgroup CYCLEATTRS noautolegend ;;

***code from above;**

**xAXIS VALUES=(1 to 35 BY 5) label='Age'**

**labelattrs=(family=arial weight=bold);**

**yaxis values=(3 to 14 by 1)**

**Label="Aggregated Data Based on Age Participated"**

**labelattrs=(family=arial weight=bold);;**

**y2axis values=(3 to 14 by 1) display=none Label="Rates"**

**display=(noticks novalues) labelattrs=(family=arial**

**weight=bold) LABELPOS=top;**

**yaxistable age_inj_rate rate_ss rate_ms / position=right**

**location=outside valueattrs=(color=black weight=bold);**

**run**;

Step 4: Add Total Population Data Summary


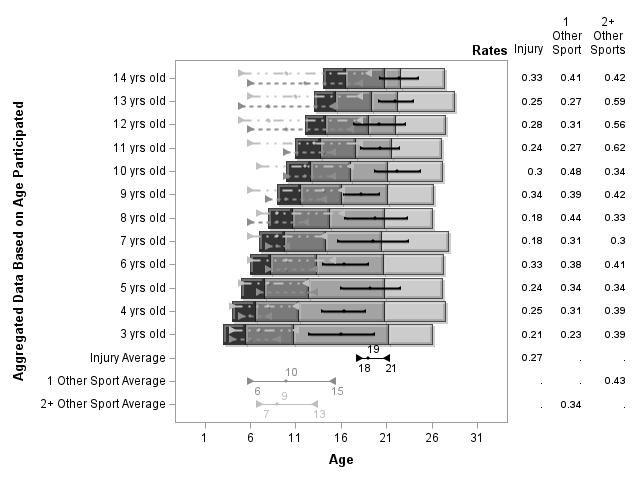


ods graphics on /noborder;

**proc** **sgplot** data=work.sgroup CYCLEATTRS noautolegend ;;

***code from above;**

xAXIS VALUES=(**1** to **35** BY **5**) label='Age'

labelattrs=(family=arial weight=bold);

yaxis values=(**0** to **14** by **1**)

Label="Aggregated Data Based on Age Participated"

labelattrs=(family=arial weight=bold);

y2axis values=(**0** to 14 by 1) display=none Label="Rates"

display=(noticks novalues) labelattrs

(family=arial weight=bold) LABELPOS=top;

yaxistable age_inj_rate rate_ss rate_ms / position=right

location=outside valueattrs=(color=black weight=bold);

**run**;
